# The pH-Responsive Liposomes—The Effect of PEGylation on Release Kinetics and Cellular Uptake in Glioblastoma Cells

**DOI:** 10.3390/pharmaceutics14061125

**Published:** 2022-05-25

**Authors:** Eirik A. L. Rustad, Susannah von Hofsten, Robin Kumar, Eirik A. Lænsman, Gerd Berge, Nataša Škalko-Basnet

**Affiliations:** 1 Drug Transport and Delivery Research Group, Department of Pharmacy, UiT The Arctic University of Norway, 9019 Tromsø, Norway; eirik.a.rustad@uit.no (E.A.L.R.); rku014@uit.no (R.K.); 2 Tumor Biology Research Group, Department of Medical Biology, UiT The Arctic University of Norway, 9019 Tromsø, Norway; susannah.hofsten@uit.no (S.v.H.); eirik.a.lansman@uit.no (E.A.L.); gerd.berge@uit.no (G.B.)

**Keywords:** pH-responsive liposomes, PEGylated liposomes, glioblastoma, calcein, cellular uptake

## Abstract

Nanomedicine has been, to a certain degree, a success story in the development of superior anticancer therapies. However, there are tumors that remain a huge challenge for nanoformulations, for instance, brain tumors such as glioblastoma, the most common and aggressive brain tumor. To utilize the fact that such tumors are characterized by an acidic extracellular environment, we selected pH-responsive liposomes as a potential drug delivery system for superior delivery to GBM. Liposomes comprising PEGylated lipid of two chain lengths with encapsulated fluorescent marker calcein were characterized and challenged against non-PEGylated vesicles. The in vitro calcein release from three liposomal formulations (<200 nm), namely non-PEGylated (pH-Lip) and PEGylated, pH-Lip–PEG750, and pH-Lip–PEG2000, was followed at three pH conditions to prove the pH-responsiveness. The intracellular delivery of a liposomally encapsulated marker was determined in GL261 glioblastoma cell lines in vitro using both flow cytometry and confocal microscopy. The inclusion of PEG2000 within liposomal formulation resulted in reduced in vitro pH-responsiveness compared to pH-Lip and pH-Lip750. All three pH-responsive liposomal formulations improved intracellular uptake in GL261 cells compared to non-pH-responsive liposomes, with negligible differences regarding PEG length. The proposed formulations should be further evaluated in glioblastoma models.

## 1. Introduction

Nanoparticles have been proposed as a drug delivery system targeting glioblastoma due to their potential to specifically deliver encapsulated drugs to the desired targets by improving efficacy and reducing unwanted biodistribution, as compared to free drugs [1,2]. Most drugs lack the physicochemical properties and specificity to successfully enter the brain from systemic circulation, limiting their ability to reach the glioblastoma tumor. Liposomes have been among the most investigated nanoparticle drug delivery system due to their biocompatibility, low toxicity, ability to load both hydrophilic and hydrophobic drugs, and surface modification. Although great progress has been made, current cancer-targeted liposomes in clinical practice have limitations in treating glioblastoma (GBM) [3,4,5]. This is highly unfortunate since GBM is one of the most aggressive types of tumors affecting the brain and an increasing problem globally [6,7]. Although temozolomide was the first substance that, combined with radiotherapy, improved overall survival, the median survival still remains just over one year [8,9]. Treatment limitations for GBM arise from the high genetic and phenotypic heterogeneity of the tumor, drug resistance, and the tumor’s location behind the blood–brain barrier (BBB) [7].

The BBB is a critical obstacle of entry to the brain [10,11]. This highly selective neuroprotective barrier protects the central nervous system (CNS) from potentially harmful pathogens. For the brain to maintain homeostasis, transport of all solutes, synaptic activities, and oxygen delivery are maintained by multiple cell types within the neurovascular unit (NVU), limiting efficient drug delivery [12]. Moreover, the blood–brain tumor barrier (BBTB) is a damaged or undeveloped NVU that leads to leakage and pooling of macromolecules. This mechanism has long been one of the major incentives for the benefits of using nanoparticles for drug delivery. However, clinical trials have indicated that the limitations of current approaches remain [3,4,13]. Trying to understand the reasons behind disappointing clinical outcome, Brown and coauthors studied the effects of nanoparticle composition, size, shape and stiffness on ability to penetrate across BBB, concluding that the particle composition impacts the penetration to the greatest extent [14]. However, for liposomes, one of the, arguably, most highly relevant issues remains the limited control over drug release, often resulting in suboptimal amounts of drug reaching tumor tissue [15]. The need to focus on a nanoformulation’s ability to release the drugs in a responsive manner has never been more evident. Sometimes, it is beneficial to go “back” and investigate all steps in optimization of the nanocarriers in vitro, to gain better insight and predict the behavior in vivo.

It is not only the ability to release the drug in a predictable and responsive manner; the surface characteristics of a nanocarrier should be exploited to optimize both the nanoparticle’s circulation time as well as drug release at targeted sites [16]. Polyethylene glycol (PEG) on the liposomal surface has been a well-established strategy for improving the effectiveness of liposomal formulations through prolonged circulation time. By reducing nanoparticles’ clearance by the mononuclear phagocyte system (MPS), the liposomes gain increased circulation time and stability. However, recent publications debate PEG’s role in cell interactions and drug release from liposomes [15,17].

In the present work, we have focused on two main liposomal features, attempting to understand their interplay to optimize drug delivery. Utilizing the acidity of the extracellular environment surrounding glioblastoma as a possible drug release trigger [18,19], we developed pH-responsive liposomes able to release the model marker calcein in a pH-responsive manner. Addressing the debate of Pegylation versus non-Pegylation [20], we compared the PEGylated pH-responsive liposomes of two chain lengths (750 and 2000, respectively) with non-PEGylated pH-responsive vesicles for their calcein release pattern and cellular fate. The GL261 glioblastoma cells served as a tumor model, and cellular uptake on liposomal calcein was followed in vitro by both flow cytometry and confocal microscopy [21].

## 2. Materials and Methods

### 2.1. Materials

Reagents and chemicals used were of analytical grade and are commercially available. Specifically, dioleoylphosphatidylethanolamine (DOPE), distearoylphosphoethanolamine polyethylene glycol 750 (DSPE-PEG750), distearoylphosphoethanolamine polyethylene glycol 2000 (DSPE-PEG2000), and cholesteryl hemisuccinate (CHEMS) were purchased from Avanti Polar Lipids, Inc. (Alabaster, AL, USA). Lipoid S 100 (>94% soybean phosphatidylcholine, PC) was a generous gift from Lipoid GmbH (Ludwigshafen, Germany). Wheat germ agglutinin (CF^®^640R WGA) for cell membrane staining was obtained from Biotium, Inc. (San Francisco, CA, USA). The fluorescent marker calcein was purchased from Sigma-Aldrich (St. Louis, MO, USA). The analytical reagents and solvents were purchased from Sigma-Aldrich.

### 2.2. Liposome Preparation

The thin film method used to prepare pH-responsive liposomes has been described previously [22]. In brief, the DOPE, CHEMS, and DSPE-PEG750 or DSPE-PEG2000 (molar ratio of 6:4 and 6:4:0.1, respectively) were dissolved in a 50:50 methanol:chloroform (*v*/*v*) solvent mixture in a round bottom flask. The solvents were removed under reduced pressure on a Büchi Rotavapor R-114 with vacuum pumpV-500 (Büchi Labortechnik, Flawil, Switzerland) at 45 °C and 60 rpm to obtain a thin lipid film that was left to completely dry at 50 mbar for 1 h. The dry lipid film was hydrated with a buffered solution of 80 mM calcein for the calcein containing liposomes or PBS (pH 7.4) for the non-calcein containing liposomes. The liposomes were stepwise hand extruded through Nucleopore^®^ polycarbonate membranes (0.8, 0.4, 0.2, and 0.1 µm pore sizes, three cycles for each size).

For calcein containing liposomes, the extravesicular calcein was removed by ultracentrifugation at 300,000× *g* for 1 h at 10 °C (L8-70M Ultracentrifuge; Beckman Instruments, Palo Alto, CA, USA). The supernatant was carefully removed before the pellet was gently resuspended in PBS using a bench vortex mixer and centrifuged for an additional 1 h. The supernatant was then removed and the pellet gently resuspended in PBS by vortexing to obtain the final liposomal formulations.

### 2.3. Characterization of Liposomes

The mean vesicle size, polydispersity index (PDI), and zeta potential were determined using Malvern Zetasizer Nano-ZS Zetasizer (Malvern Instruments, Malvern, UK). All suspensions were diluted 1:20 in PBS to ensure stable pH and measured after 15 min equilibration time. The measurements were performed in triplicate.

Calcein entrapment was determined using spectrofluorometric analysis on a FLUOstar Galaxy (BMG LabTechnologies GmbH, Offenburg, Germany) spectrofluorometer. Briefly, calcein-free liposomes were disrupted by diluting liposomal formulations in buffer containing Triton-X (final concentration 1%) and thoroughly mixed on a vortex mixer before analysis. Calcein concentrations, both for liposomes and unentrapped calcein, were then determined spectrophotometrically.

### 2.4. In Vitro pH-Responsive Calcein Release

The pH-responsive release was determined using spectrofluorometric analysis on FLUOstar Galaxy (BMG LabTechnologies GmbH, Offenburg, Germany). The gain and wavelength (λ) of excitation (ex) and emission (em) were optimized and selected as λex = 485 nm, λem = 520 nm, gain = 19). Liposomes were diluted with PBS (pH 7.4) in a 1:4 volume ratio, and aliquots of 150 µL were transferred to a 96-well plate in triplicate. After time zero measurements, HCl (0.1 M) (5 and 10 µL) was added to each well to obtain the desired pH and measured for 240 min. pH was measured in each well after HCl addition and presented as average.

### 2.5. Cell Culture

Mouse glioblastoma GL261 (ACC-802, Deutsche Sammlung von Mikroorganismen und Zellkulturen) cells were maintained in Dulbecco’s Modified Eagle Medium (DMEM) (Sigma-Aldrich) supplemented with 10% (*v*/*v*) fetal bovine serum (FBS) (Sigma-Aldrich). The cells were incubated at 37 °C in a humidified environment of 5% CO_2_.

### 2.6. Cell Viability

Cell viability of the cells exposed to different liposomal formulations and calcein was determined using the CellTiter 96^®^ AQ_ueous_ One Solution Cell Proliferation Assay (Promega, Madison, WI, USA). Cells were seeded at 1 × 10^4^ cells/well in a 96-well plate and incubated overnight. DMEM supplemented with 10% FBS was used to pre-dilute all liposomal formulations right before treating the cells. The cell medium was carefully removed and 100 µL of fresh cell medium (negative control), 1% Triton-X (positive control), and three concentrations of liposomes (10, 25, and 50 µg/mL of lipids, respectively) were added and incubated at 37 °C with 5% CO_2_. At 1 h before endpoints, 20 µL of pre-warmed CellTiter solution was added to each well and incubated until endpoints of 4 and 24 h, respectively. The 96-well plate was mixed for 5 s prior to measuring absorbance at 490 nm in a microplate reader (VersaMax Tunable Microplate Reader; Molecular Devices, LLC) [23].

### 2.7. Cellular Uptake

The time-dependent cellular uptake of calcein was quantified by flow cytometry (BD Accuri™ C6 Plus Flow Cytometer; BD Biosciences, San Jose, CA, USA). GL261 tumor cells were seeded on 24-well plates (300 µL, 1 × 10^5^ cells/well) and incubated for 24 h prior to treatment. The medium was exchanged in all wells with 1 mL of DMEM with 10% FBS or DMEM with 10% FBS containing pH-Lip, pH-Lip750, pH-Lip2000, or Lip. We incubated all calcein containing liposomes for 2 and 24 h. The cells were washed with PBS and harvested from the plates using Trypsin-EDTA solution (Sigma), followed by a resuspending step in DMEM with 10% FBS. The cell suspensions were centrifuged (300 rpm, 5 min) and the pellets resuspended in 300 µL PBS [24].

Confocal microscopy was performed to confirm the intracellular uptake of calcein. GL261 cells were seeded in 8-well 1,5# borosilicate plates with coverslips and pre-treated with fibronectin (3 × 10^4^ cells/well) 24 h before treatment. Cells were incubated with pH-Lip, pH-Lip750, pH-Lip2000, or Lip for 4 h. The cell membrane was labeled with 1:100 WGA640 for 10 min. The confocal images were acquired using a Zeiss LSM 780 Confocal Microscope.

### 2.8. Statistical Analysis

Data are presented as the mean ± standard deviation (SD). One-way analysis of variance (ANOVA) was used to determine significance, after which *post-hoc* Bonferroni correction was used for comparison between individual groups. Statistical significance was established at *p* < 0.05.

## 3. Results

### 3.1. Liposomal Charcateristics

The vesicle size, polydispersity index (PDI), and zeta potential of the liposomal formulations are shown in Table 1. The particle size of calcein loaded pH-responsive liposomes was between 160 and 170 nm with a PDI under 0.2, indicating rather homogeneous vesicle populations. As determined by zeta potential measurements, DOPE:CHEMS liposomes exhibited a net negative charge due to the amphiphilic stabilizer CHEMS, in agreement with previous findings [17]. The presence of calcein affected the surface charge; however, the overall charge remained negative. Non-pH-responsive liposomes were neutral or close to neutral, as expected. Regarding the effect of PEG molecules on liposomal bilayers, it was evident that hydrophilic PEG molecules reduced the surface charge. Moreover, it was confirmed that the surface charge reduction was PEG chain-dependent: the longer the PEG chains, the more pronounced was surface charge reduction that was observed, regardless of the calcein presence in liposomes. This shielding effect of PEG observed in our study was in accordance with literature [25].

Entrapment efficiency for calcein was found to be between 0.2 and 0.5% for all liposomal formulations, more specifically, for Lip_C, it was 0.21%, 0.32% for pH-Lip_C, 0.20% for pH-Lip–PEG750_C, and for pH-Lip–PEG2000_C 0.45%, respectively. The entrapment can be considered low and was probably affected by extensive filtration and washing of liposomal pellet prior to entrapment determination. However, our aim was to ensure that free calcein was removed from the surrounding medium while the calcein concentration within liposomes remained high enough to proceed with in vitro release studies. Calcein concentration within liposomes was between 0.2 and 0.4 mM, sufficiently high to follow the release of calcein.

### 3.2. The pH-Responsive Calcein Release

**Figure 1:**
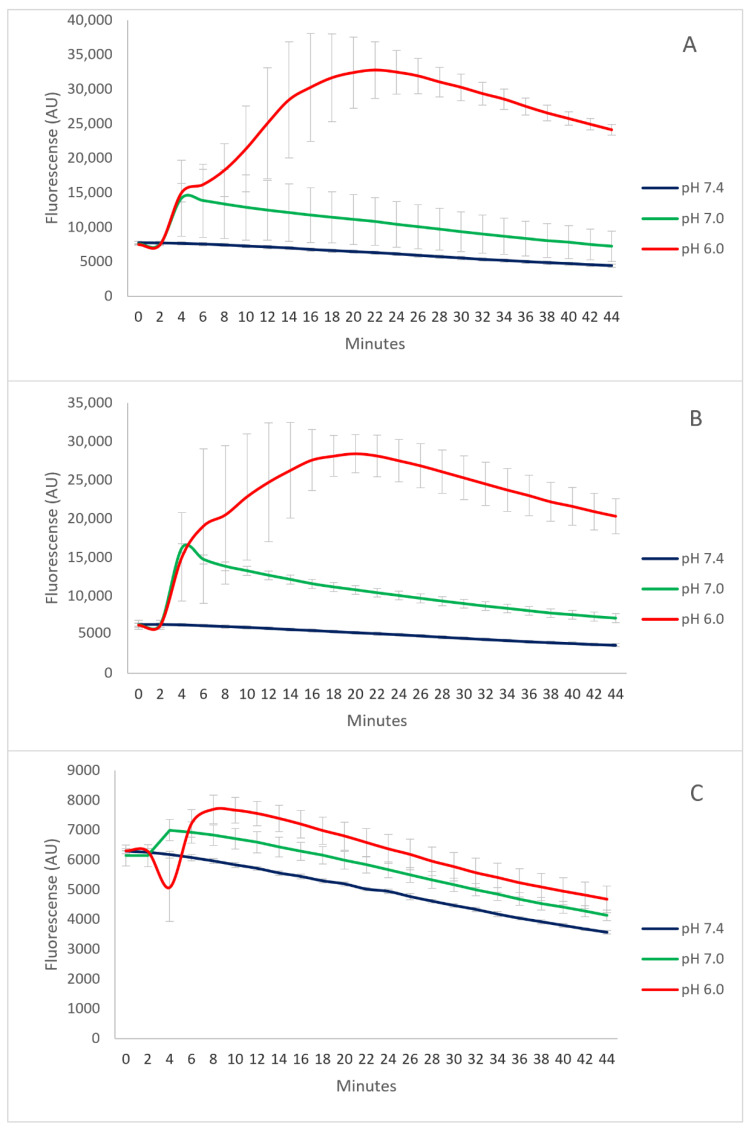
The pH-dependent calcein release from pH-Lip, pH-Lip–PEG750 and pH-Lip–PEG2000 after the addition of HCl to a 7.4 PBS buffer medium. Data represent the mean ± standard deviation (n = 3). (**A**): pH-Lip (**B**): pH-Lip–PEG750 and (**C**): pH-Lip–PEG2000.

The calcein fluorescence is self-quenching at a calcein concentration of 80 mM [17]. Therefore, this starting concentration was used for incorporation into the liposomes to determine the pH-responsive release. Despite rather low entrapment efficacy, the calcein release was successfully followed. The release of calcein from all liposomal formulations was rapid upon the addition of HCl (Figure 1). The pH-Lip–PEG750 (Figure 1B) reached a release plateau after 20 min at pH 6.0, with no significant difference in calcein release compared to non-PEGylated pH-Lip (Figure 1A). The presence of PEG750 only reduced peak calcein release by approximately 10–15% compared to the non-PEGylated, indicating a rather small stabilizing effect. In comparison, pH-Lip–PEG2000 (Figure 1C) exhibited significantly less calcein release compared to both pH-Lip and pH-Lip–PEG750, confirming the stabilizing effect of the longer PEG chain compromising the pH-responsiveness.

At pH 7.4, we did not observe calcein release from any tested formulations. This finding suggests that pH-responsive liposomes indeed retained entrapped calcein at physiological conditions. The fluorescence loss observed after the time point of the release plateau (Figure S1) can be explained by photobleaching. In addition, the direct comparison of the release from different formulations at the same pH is presented in Figure S2.

### 3.3. Cellular Uptake

**Figure 2:**
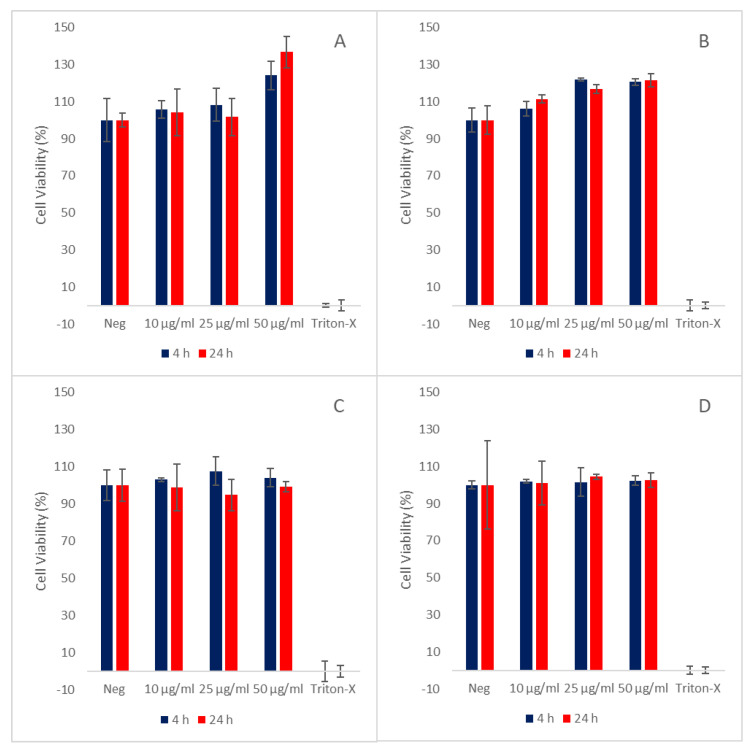
The GL261 cell viability assessed by MTS assay performed at 4 and 24 h incubation with different liposomal formulations. Data given as mean ± SD of % viability (n = 3). (**A**): pH-Lip, (**B**): pH-Lip750, (**C**): pH-Lip2000, (**D**): Lip. DMEM with 10% FBS used as negative control (Neg) and 0.1% Triton-X as positive control. pH-Lip: pH-responsive liposomes without PEG, pH-Lip–PEG750: pH-responsive with DSPE-PEG750, pH-Lip–PEG2000: pH-responsive with DSPE-PEG2000, Lip: non-pH-responsive, non-PEGylated liposomes.

Prior to the cellular uptake experiments, we wanted to confirm that the liposomal formulations did not exhibit any cytotoxic effects. Therefore, the viability of GL261 cells was examined using the MTS assay [23]. As shown in Figure 2, normal cell growth was not inhibited in any cells exposed to the liposomal formulations. Therefore, the pH-Lip (Figure 2A), pH-Lip–PEG750 (Figure 2B), pH-Lip–PEG2000 (Figure 2C), and Lip (Figure 2D) did not appear to exhibit any clear cytotoxic effects.

**Figure 3:**
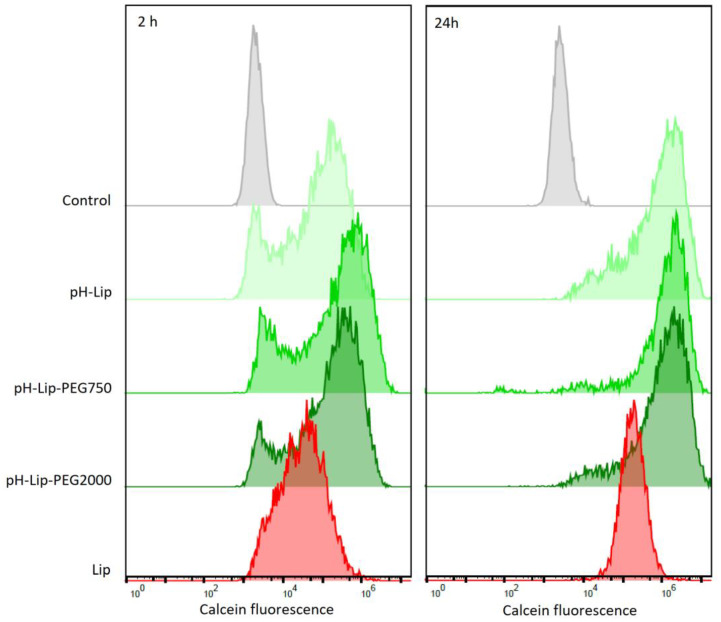
Flow cytometry histograms of calcein fluorescence in GL261 after 2 and 24 h incubation with different liposomal formulations. Control is DMEM + 10% FBS. pH-Lip: pH-responsive liposomes without PEG, pH-Lip–PEG750: pH-responsive with DSPE-PEG750, pH-Lip–PEG2000: pH-responsive with DSPE-PEG2000, Lip: non-pH-responsive, non-PEGylated liposomes.

The uptake of calcein in GL261 glioblastoma tumor cells from calcein-containing formulations was analyzed using flow cytometry (Figure 3). Rather surprisingly, all pH-responsive liposomal formulations increased calcein uptake in glioblastoma cells compared to free calcein uptake, regardless of liposomal compositions. Neither PEG nor PEG chain length affected the calcein uptake (Figure 3), contrary to a recent publication [15]. All pH-responsive liposomal formulations exhibited increased calcein uptake compared to non-pH-responsive phosphatidylcholine (PC) liposomes. Similar trends could be already seen at a shorter incubation time of 2 h.

**Figure 4:**
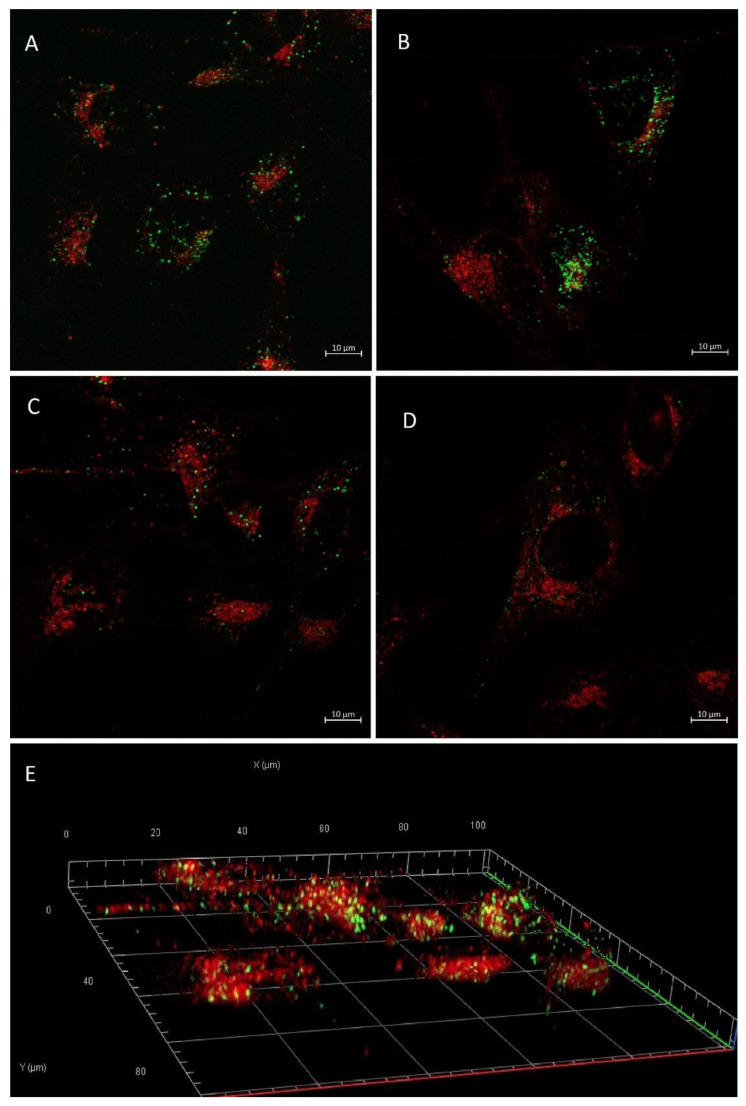
Confocal microscopy images of calcein uptake in GL261 cells after 4 h of incubation. (**A**) pH-Lip, (**B**) pH-Lip–PEG750, (**C**) pH-Lip–PEG2000, (**D**) Lip, (**E**) pH-Lip–PEG2000 in 3D representation of z-stack images. Green: calcein; Red: WGA640. pH-Lip: pH-responsive liposomes without PEG, pH-Lip–PEG750: pH-responsive with DSPE-PEG750, pH-Lip–PEG2000: pH-responsive with DSPE-PEG2000, Lip: non-pH-responsive, non-PEGylated liposomes.

The intracellular delivery of calcein was also confirmed by the direct observation of the cells using confocal microscopy. Following a 4 h liposome-cell incubation, intense green color (calcein) can be observed intracellularly (Figure 4). When comparing the confocal microscopy findings with the flow cytometry results, it is evident that the green intracellular vesicles confirm the successful delivery of calcein from all pH-responsive formulations (Figure 4A–C). All images were also evaluated as z-stack to create 3D representations (Figure 4E) to ensure that the observed calcein uptake was indeed intracellular and not a fluorescence associated with the cellular surface.

## 4. Discussion

Only a small number of drugs, such as temozolomide [26], can naturally cross the BBB due to its high level of selectivity, excluding most chemotherapeutic drugs. Nanocarrier drug delivery systems could overcome the limitations of current chemotherapeutic drugs—by improving the BBB penetration, accumulating at tumor sites, and interacting with the tumor microenvironment and tumor cells to release the chemotherapeutic drug [1,27,28]. Although various approaches have been explored to improve the fate of nanocarriers in vivo, leading to better therapy outcomes, the challenges have managed to hamper faster development in the field. It is well agreed that the accumulation of nanoformulations in tumor tissue is highly dependent on the particle size, particle properties, and surface modifications [1,27,28]. However, to be able to successfully reach and treat glioblastoma through the BBB barrier, the ability to reduce phagocytosis by the MPS, better specific targeting, and controllable drug release need to be solved [29,30]. Although the ultimate proof that nanoformulations can indeed reach/target glioblastoma would be in vivo proof-of-concept, we believe that deeper insight gained in extensive in vitro studies serves as a sound base for further evaluations.

We have focused on two important issues, namely, the ability of the nanocarrier to control the drug release at a target site, and the role Pegylation plays in both drug release as well as intracellular fate. We therefore proposed that the acidic environment linked to glioblastoma [31] could be used as both a targeting strategy and trigger for drug release. The responsive release is highly relevant when attempting to improve cancer treatment [31]. Liposomes composed of DOPE and CHEMS were chosen for our studies due to the promised outcomes of previous studies [17,32,33]. Due to the conical shape of DOPE, it does not form stable liposomes on its own. To form stable liposomes, a carboxylated lipid called CHEMS is inserted between DOPE molecules, which promotes the formation of liposomes. The DOPE:CHEMS liposomes’ pH-responsiveness arises from the protonation of CHEMS’ carboxyl group, reducing its stabilizing properties. Therefore, when these liposomes are exposed to an acidic environment, they destabilize and form inverted hexagonal micelles that release the encapsulated drug.

Prior to expanding the targeting strategy by including the relevant ligand on the liposomal surface, we focused on assuring the principles of pH-responsiveness in vitro, especially the role PEG molecules can potentially have on the release of liposomal-associated drugs or markers. Recently, the issue of PEGylation versus PEG-free nanoparticles has been extensively studied [15,17,34,35]. PEG modification is believed to provide a steric hindrance on the surface, reduce adsorption, and prolong the blood circulation time by reducing MPS uptake. In addition, there have been reports on the development of anti-PEG antibodies and repeated administration of PEGylated liposomes leading to an acceleration in blood clearance [20,35].

However, much of the research carried out regarding the role of PEGylation has been focused on relatively high PEG concentrations and mostly on PEG 2000, the most used PEG chain [3,15,20]. Our study aimed to explore how the PEG chain length affects liposomes’ pH responsiveness as well as the overall impact that the PEGylation and pH-responsiveness have on cellular uptake of a model marker in GL261 glioblastoma cells. All liposomal formulations were prepared to be of a similar size, with mean diameters <180 nm, assuring that the observed effects are not correlated to the differences in vesicle size. According to literature, the selected size was considered acceptable for tumor targeting; the vesicles were small enough to reduce MPS uptake and ensure tumor vasculature permeability [36]. One could argue that smaller vesicles could offer advantages considering the in vivo fate, however, in this in vitro optimization, we needed to ensure that the liposomes maintained a high calcein load to be able to detect even minor differences in the release pattern; moreover, we wanted to ensure detectable fluorescence during evaluation of cellular uptake by confocal microscopy. Although our in vitro release findings demonstrated that the PEG chain length of PEGylated liposomes impacted the calcein release, expecting that similar effects would be detectable for cellular uptake, that was not the case.

The presented cellular uptake data indicate that the underlying mechanism of cellular uptake involves other complex mechanisms rather than the pH-responsiveness alone. Simões et al. [32] proposed that the polar head of PE or DOPE contributes to the low hydration layer of the polar phospholipid head compared to PC. Consequently, this facilitates a favorable interaction between lipid bilayers, resulting in an increased affinity of liposomes for the cell membrane. There are several mechanisms responsible for the internalization of liposomes. Moreover, several factors influence the intracellular delivery of drugs from liposomes, such as vesicle size, surface charge, and steric stabilization of the liposomal surface [37,38]. Thus, it is rather challenging to directly compare the findings reported by various research groups working with pH-responsive liposomes, even if the cell model would be the same, which is often not the case. It is important to note that, in our case, the molar concentration of PEG was relatively low (1 mol% of PEG), both for PEG750 or PEG2000. This concentration can be considered rather modest compared to the concentrations studied by Nunes et al. [15], who used 5 mol%.

The conformed pH-responsiveness can be considered to be the first step in the optimization of liposomes as delivery systems for glioblastoma. The next step would involve the introduction of a targeting moiety that would enable enhanced glioma targeting, as proposed, for example, by Zhao et al. [39], Poustforoosh et al. [40], and Farshbaf et al. [41]. The specific targeting offered by novel targeting moieties on vesicle surfaces, together with the ability to release entrapped therapeutic cargo in response to the environmental stimuli, may offer advantages both for well-studied anticancer drugs as well as novel drug candidates. Considering the challenges that the BBB imposes on nanocarriers, the final proof-of-concept needs to involve in vivo evaluation.

## 5. Conclusions

To gain deeper insight on the role of PEGylation on the drug release and intracellular fate of pH-responsive liposomes in vitro, we focused on two different PEG chain lengths. The results of pH-responsiveness indicate that the liposomes comprising longer PEG chains (pH-Lip–PEG2000) were less responsive than non-PEGylated and shorter PEG chains comprising liposomes (pH-Lip and pH-Lip–PEG750, respectively). Contrary to this finding, all pH-responsive liposomal formulations exhibited similarly increased calcein uptake in GL261 glioblastoma cells compared to the non-pH-responsive liposomes (Lip). This proves that the role of PEGylations and PEG chain length needs to be further evaluated in other and more complex glioblastoma models, as well as ultimately in in vivo conditions.

## Figures and Tables

**Table 1:** Characteristics of liposomal formulations. Values are expressed as mean ± standard deviation. (n = 3).

Type of Liposome	Vesicle Size (nm)	PDI	ζ Potential (mV)
pH-Lip	145.0 (±0.5)	0.07 (±0.00)	−44.8 (±0.6)
pH-Lip_C	163.9 (±0.9)	0.11 (±0.01)	−37.2 (±1.6)
pH-Lip–PEG750	177.9 (±1.1)	0.16 (±0.01)	−21.0 (±1.7)
pH-Lip–PEG750_C	166.5 (±1.5)	0.16 (±0.01)	−31.9 (±1.8)
pH-Lip–PEG2000	160.6 (±1.5)	0.14 (±0.01)	−12.9 (±0.8)
pH-Lip–PEG2000_C	161.7 (±0.8)	0.12 (±0.01)	−16.1 (±0.8)
Lip	156.4 (±2.0)	0.14 (±0.01)	0.08 (±0.7)
Lip_C	160.1 (±0.6)	0.09 (±0.01)	4.29 (±0.9)

pH-Lip: pH-responsive liposomes without PEG, pH-Lip–PEG750: pH-responsive with DSPE-PEG750, pH-Lip–PEG2000: pH-responsive with DSPE-PEG2000, Lip: non-pH-responsive, non-PEGylated liposomes. C is indicating calcein containing formulations. PDI: Polydispersity index.

## Data Availability

Data are contained within the article or Supplementary Material.

## Supplementary Materials

The following supporting information can be downloaded at: https://www.mdpi.com/article/10.3390/pharmaceutics14061125/s1. Figure S1: Prolonged pH-responsive calcein release 4 h; Figure S2: Comparison of pH-dependent calcein release at specific pH.
