# Supplementary material for: The pH-Responsive Liposomes—The Effect of PEGylation on Release Kinetics and Cellular Uptake in Glioblastoma Cells
Source: Pharmaceutics. 2022 May 25;14(6):1125. doi: 10.3390/pharmaceutics14061125 (PMC9227832; doi:10.3390/pharmaceutics14061125)
Supplement: Supplementary file 1 [file pharmaceutics-14-01125-s001.zip › pharmaceutics-1701917-supplementary.pdf]

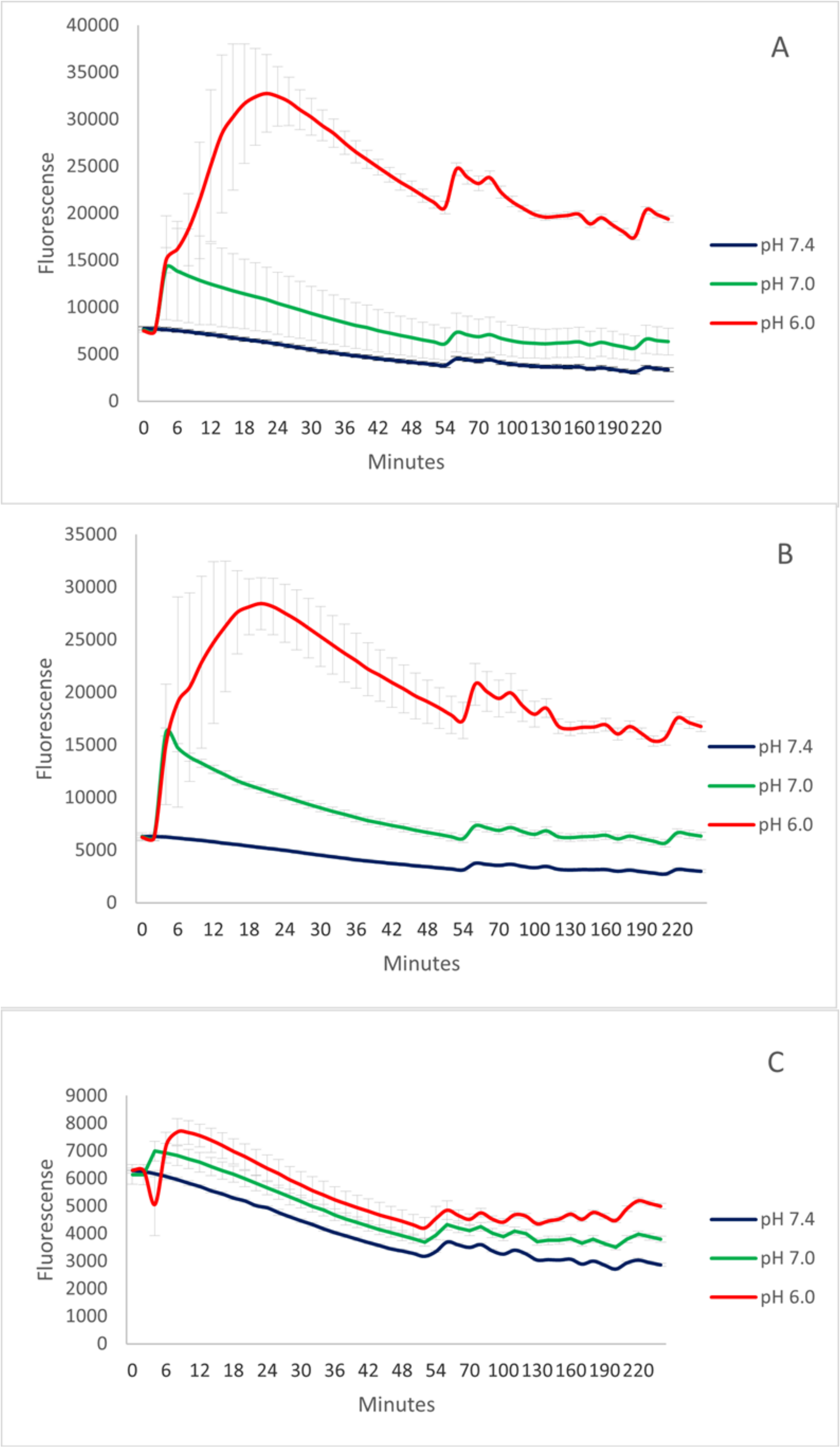

Figure S1: Prolonged pH-dependent calcein release from Ph-Lip, Ph-Lip-PEG750 and Ph-Lip-PEG2000 after addition of HCl to a 7.4 PBS buffer medium. Data represents the mean ± standard deviation (n = 3). A: Ph-

Lip B: Ph-Lip-PEG750 and C: Ph-Lip-PEG2000. pH-Lip: pH-responsive liposomes without PEG, pH-Lip-PEG750: pH-responsive with DSPE-PEG750, pH-Lip-PEG2000: pH-responsive with DSPE-PEG2000, Lip: non-pH-responsive, non-PEGylated liposomes.

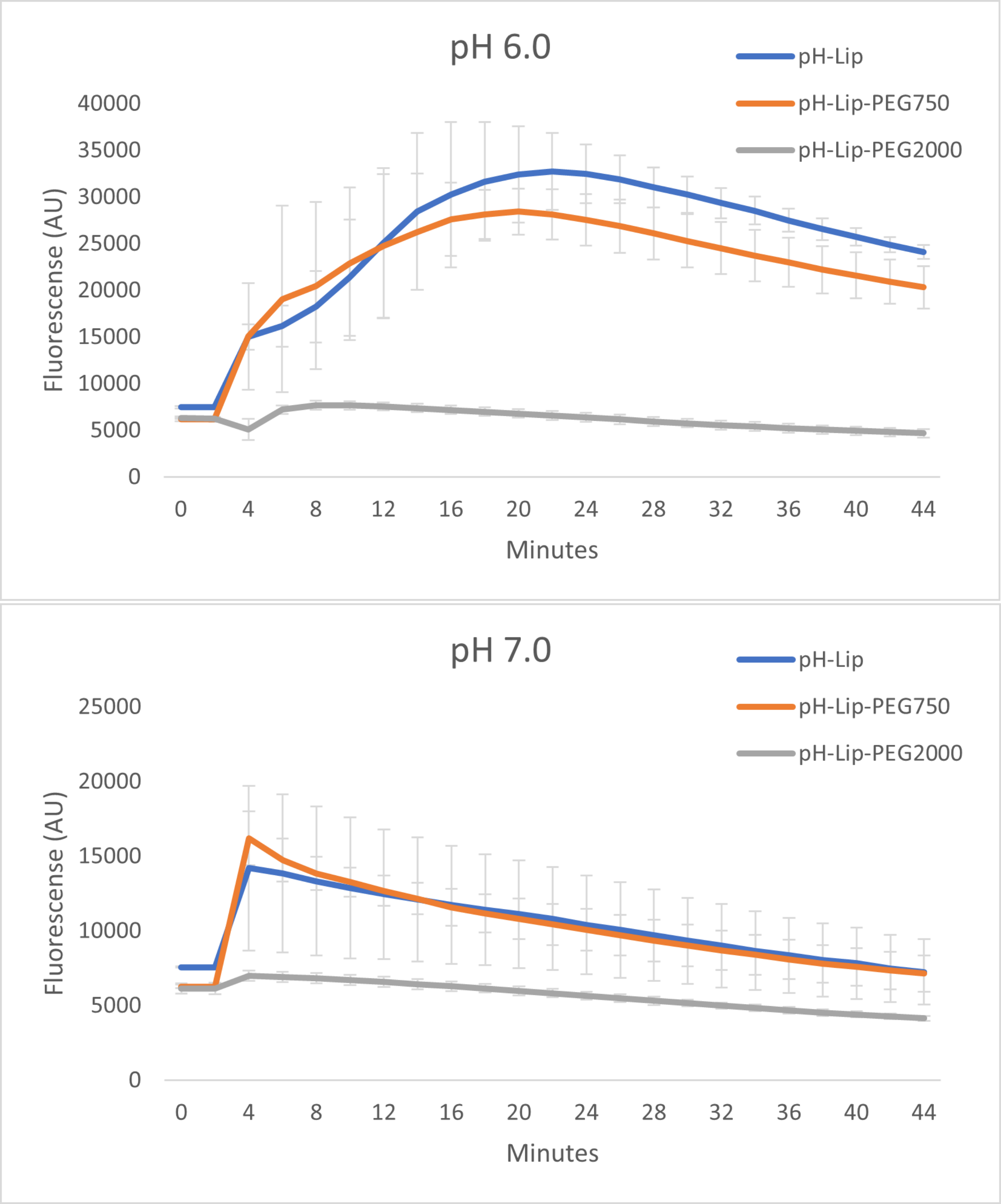

Figure S2: Comparison of pH-dependent calcein release at specific pH. Data represents the mean ± standard deviation (n = 3). pH-Lip: pH-responsive liposomes without PEG, pH-Lip-PEG750: pH-responsive with DSPE-PEG750, pH-Lip-PEG2000: pH-responsive with DSPE-PEG2000.
